# Outcomes in laparoscopic versus robotic-assisted surgery for median arcuate ligament syndrome: a systematic review and meta-analysis

**DOI:** 10.1007/s11701-026-03551-x

**Published:** 2026-06-19

**Authors:** Zahra Arabi, Georgios Geropoulos, Pasan Jayawardena, Seiver Karim, Lillian Reza, Nicola Colucci, Vanash Patel

**Affiliations:** 1 Department of Surgery and Cancer, Imperial College London, St Mary’s Hospital, 10th Floor Queen Elizabeth Queen Mother Building, W2 1NY London, England, UK; 2 Department of Surgery, Anaesthetics and Cancer, West Hertfordshire Teaching Hospitals NHS Trust, Watford General Hospital, Vicarage Road, WD18 0HB Watford, UK; 3 Addenbrookes Hospital, Cambridge University Hospitals NHS Foundation Trust, Hills Rd, CB2 0QQ Cambridge, England, UK; 4 Medical School, University College London, Gower Street, WC1E 6BT London, England, UK

**Keywords:** Median arcuate ligament syndrome, Perioperative outcomes, Systematic review, Meta-analysis

## Abstract

Median Arcuate Ligament Syndrome (MALS) is a rare condition caused by external compression of the coeliac artery by the median arcuate ligament (MAL), leading to postprandial abdominal pain, nausea, vomiting, and weight loss. This study aims to compare outcomes of robotic (RMALR) and laparoscopic (LMALR) MAL release. A systematic search of PubMed/MEDLINE, Scopus, and Embase databases was performed in accordance with the Preferred Reporting Items for Systematic Reviews and Meta-Analyses guidelines to compare perioperative and postoperative outcomes of LMALR versus RMALR. Comparative studies including adult patients undergoing either approach were included. Outcomes assessed were conversion to open surgery, resolution of pain, length of admission, postoperative complications, symptom recurrence and time to recurrence. Five retrospective comparative studies comprising 158 patients were included. No differences were found between laparoscopic and robotic approaches in conversion to open, resolution of pain, postoperative complications, length of admission, or overall time to recurrence. However, RMALR showed a significantly longer time to symptom recurrence (9.04 months, 95% CI 4.18–13.89; *p* = 0.0003). Both LMALR and RMALR are safe and effective for MALS, with comparable perioperative and postoperative outcomes. The longer symptom-free interval after robotic surgery may reflect greater precision in dissection around the coeliac axis. However, further prospective studies with standardised follow-up are needed to validate these findings.

**Supplementary Information:**

The online version contains supplementary material available at 10.1007/s11701-026-03551-x.

## Introduction

Median arcuate ligament syndrome (MALS), also known as Dunbar or coeliac artery compression syndrome, is a rare disorder with a prevalence of approximately 2 per 100,000 people [1]. It has been reported that females are affected four times more than males [2]. The underlying pathology originates by the median arcuate ligament (MAL) extrinsically compressing the coeliac artery. The MAL demonstrates a fibrous arch which joins both the left and right diaphragmatic crura. It is anatomically found at T12/L1, where it forms the aortic hiatus. Inferior to the MAL is an aorta branch named the coeliac artery. Adjacent to the coeliac artery lies the coeliac plexus, which is formed of sympathetic and parasympathetic nerve fibres.

MALS may occur when the median arcuate ligament is positioned inferior to its typical point of insertion or when the coeliac artery originates superior to its typical point of branching from the abdominal aorta [3].

In MALS, the compression of the coeliac artery may cause intermittent mesenteric ischaemia, likely being the reason for epigastric abdominal pain [2]. This diagnosis, considered as one of exclusion, commonly presents with other symptoms including weight loss, nausea, postprandial pain, vomiting and diarrhoea [4]. An important confounder to consider is that anatomical compression does not necessarily correlate with clinical symptoms, as 10–24% of the population have a low MAL insertion yet remain asymptomatic [5].

Since first described by Dunbar in 1965, the surgical management of MALS has evolved from open surgery to minimally invasive laparoscopic approach and, more recently, to robotic-assisted surgery [6]. Despite the increasing utilisation of robotic-assisted surgery, comparative evidence evaluating postoperative outcomes between laparoscopic and robotic approaches remains limited. This paper aims to compare and evaluate postoperative outcomes following LMALR and RMALR.

## Methods

### Overview

This study aims to compare outcomes of robotic and laparoscopic treatment of MALS. This study was performed according to the Preferred Reporting Items for Systematic Reviews and Meta-Analyses guidelines and the Cochrane Handbook for Systematic Reviews of Interventions [7, 8]. Title and abstract screening were performed independently by ZA and GG. Full-text review and extraction of data were performed independently by ZA and GG. Senior authors were involved for any disagreements at any point in the screening process. This study was registered on PROSPERO (CRD420261343781) [9].

### Search strategy

The PubMed/MEDLINE, Scopus, and Embase were searched for studies comparing minimally invasive median arcuate ligament release in adult patients with MALS [7]. The final search was conducted on 20th March 2026. Search terms combined condition-related keywords, including “median arcuate ligament syndrome”, “celiac artery compression syndrome”, “celiac compression syndrome”, “MALS”, and “CACS”, with intervention-related terms including “laparoscopic”, “surgery”, “management”, “treatment”, and “operative”. The detailed search strategy for each database is provided in Supplementary Table 1. The references of included studies were also reviewed for any potential relevant studies.

### Study selection

Comparative studies reporting outcomes of both laparoscopic median arcuate ligament release (LMALR) and robotic median arcuate ligament release (RMALR) in adult patients with confirmed MALS were deemed eligible for our analysis. At our search the following studies were excluded: (1) studies published in non-English language; (2) conference abstracts, case reports, editorials, and conference posters; (3) unpublished manuscripts; (4) studies including paediatric patients; and (5) studies not reporting comparative outcomes between laparoscopic and robotic cohorts. No restrictions were placed on publication date or sample size.

### Risk of bias

The quality assessment was performed independently by ZA and GG through the use of the Methodological Index for Non-Randomised Studies (MINORS) tool [10]. A senior author was consulted in case of disagreements. Each study was evaluated across the 12 domains applicable to comparative studies, which directly compared robotic and laparoscopic approaches. MINORS domains were scored as if not reported (0), if reported but inadequate (1), or if reported and adequate detail provided (2). A MINORS score of less than 12 is considered a high risk of bias, while MINORS scoring between 13 and 18 is considered a moderate risk, and 19–24 to be low risk. The GRADE assessment was also used to evaluate the certainty of key outcomes [11].

### Data extraction and outcomes

Data extracted from each study by two reviewers, independently, and included publication details, study design, enrolment period, sample size, treatment-arm size, sex distribution, age, and baseline clinical characteristics. Additional extracted variables included body mass index, preoperative and postoperative peak systolic velocity (PSV), operative time, length of hospital stay, follow-up duration, postoperative symptom status, symptom recurrence, time to recurrence, conversion to open surgery, postoperative complications, and reinterventions where available.

The primary outcomes of interest were symptom recurrence, time to recurrence, conversion to open, postoperative complications, length of hospital stay, and symptom resolution. Secondary outcomes included postoperative symptom profiles, PSV, and reintervention where reported. Outcome definitions were extracted as reported in the original studies. Where definitions or follow-up intervals differed across studies, findings were interpreted with caution, and this was considered during synthesis.

### Statistical analysis

Dichotomous outcomes were summarised using odds ratios (ORs) with 95% confidence intervals (CIs), and continuous outcomes using mean differences (MDs) with 95% CIs. *P*-values < 0.05 were considered statistically significant. Statistical heterogeneity reflected by the I², with values of I² >50% considered indicative of substantial heterogeneity. A fixed-effects model was used when statistical heterogeneity was low, and a random-effects model was used where heterogeneity was substantial. Outcomes were pooled only when reported with sufficient numerical detail. Where quantitative synthesis was not possible because of limited reporting or heterogeneity in outcome definitions, findings were summarised narratively. Meta-analysis plots were formulated using Review Manager 5.

## Results

### Literature search

**Fig. 1:**
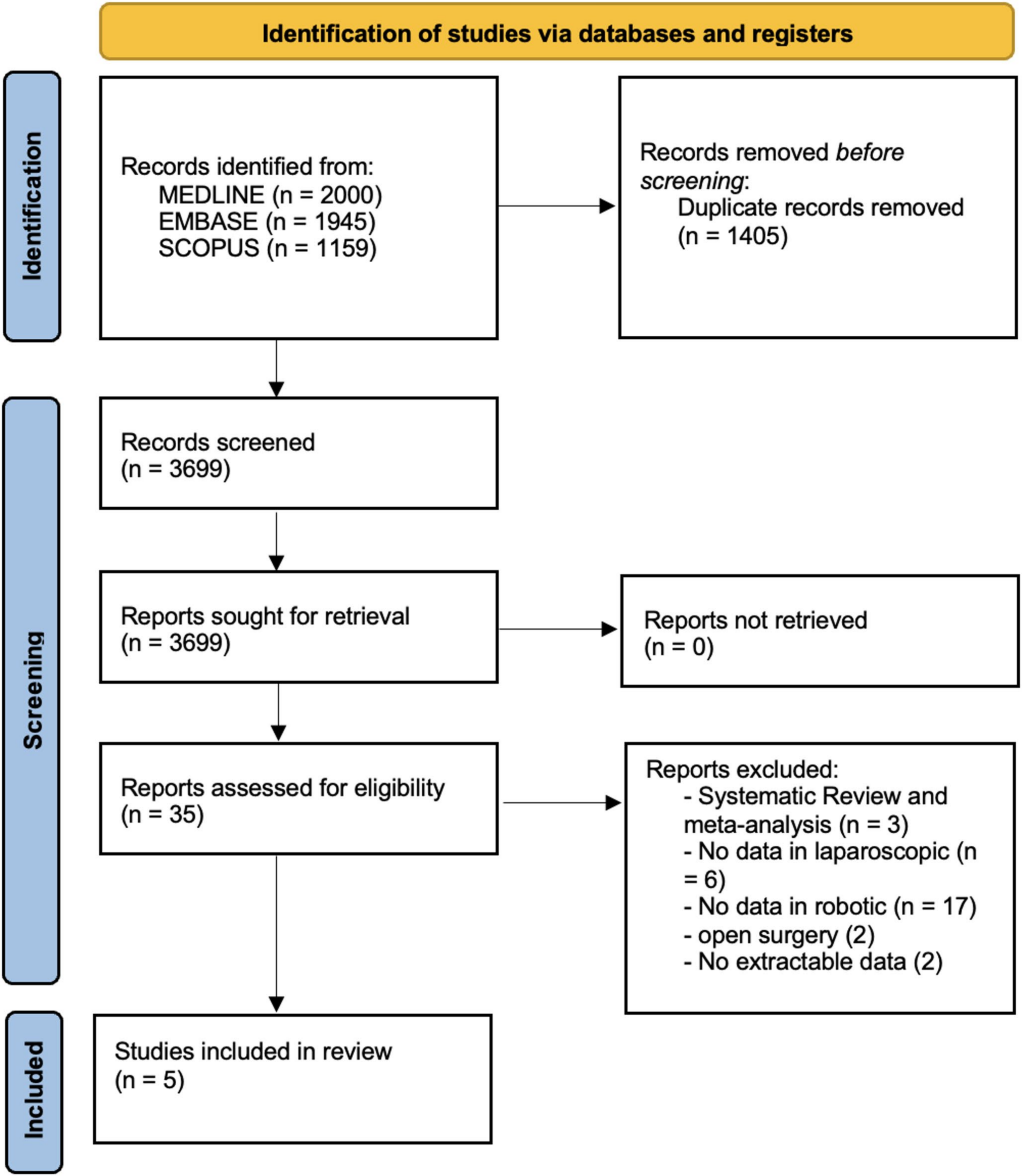
PRISMA flowchart

Literature search yielded 5104 articles. Following de-duplication (1405), 3699 articles were available for screening (Fig. 1). 35 studies were included for full-text review. Full-text analysis resulted in five studies which met inclusion criteria. Table 1 summarises the key characteristics of the five included retrospective studies.

**Table 1:** Basic characteristics of the included studies

Author (Year)	Country	Enrolment Dates	Total patients (*n*)	Total lap (*n*)	Total robotic (*n*)	Female (%)	Age in lap group (mean ± SD)	Age in robotic group (mean ± SD)
Butz et al. (2024) [12]	Germany	2014–2023	20	3	17	70	36	34
Khrucharoen et al. (2019) [13]	US	1999–2018	34	16	18	76	41.5	38.5
Do et al. (2013) [14]	US	2006–2012	16	12	4	63	46.1 ± 17.3	51.8 ± 25.3
Fay et al. (2025) [15]	US	2015–2023	38	27	11	76	38.8 ± 14.2	41.1 ± 17.9
Shin et al. (2021) [16]	US	2018–2019	50	24	26	96	34.9 ± 15	37.5 ± 17.3

### Baseline patient characteristics

Diagnosis of MALS was carried out through a combination of clinical symptoms, exclusion of other differentials and forms of vascular imaging proving coeliac artery compression. Duplex ultrasonography (DUS) was the primary diagnostic modality in all studies, with diagnosis supported by elevated expiratory coeliac artery PSV > 200 cm/s, consistent with haemodynamically significant extrinsic compression. This was used as an objective diagnostic criterion across studies [12–16].

In terms of radiological examinations, Computed tomography angiography (CTA) or magnetic resonance angiography (MRA), was often used in conjunction with DUS to confirm anatomical coeliac artery compression. In all cohorts, patients underwent extensive preoperative investigations, including oesophago-gastro-duodenoscopy to exclude alternative causes of abdominal pain [12, 13, 15, 16].

The laparoscopic approach for MAL release involved five ports, including a supra- or peri-umbilical camera port, bilateral upper-quadrant working ports, and a subxiphoid port for liver retraction. Dissection was performed through the gastrohepatic ligament, with division of the MAL and surrounding fibrous tissue to achieve complete exposure and skeletonisation of the coeliac artery at its origin [12, 14].

The robotic-assisted approach followed similar anatomical principles but involved five to six ports. Of the five studies, three had specified the robotic platform used, which were all da Vinci Surgical System (Intuitive Surgical, Sunnyvale, CA, USA) [13, 14, 16].

Duration of symptoms prior to surgery ranged from 12 to 24 months, with no consistent differences between laparoscopic and robotic cohorts [13, 16]. This prolonged preoperative course highlights the extensive diagnostic evaluation typically undertaken before surgical management is pursued.

Pre-operative symptoms were collected across the five studies and summarised in Table 2. Inclusion and exclusion criteria are summarised in Table 3.

**Table 2:** Pre-operative symptoms reported as n (%) and % rounded to nearest whole number

Author (Year)	Abdominal pain Lap	Abdominal pain Robotic	Nausea/ vomiting Lap	Nausea/ vomiting Robotic	Diarrhoea Lap	Diarrhoea Robotic	Weight loss Lap	Weight loss Robotic
Butz et al. (2024) [12]	3 (100)	15 (88)	1 (33)	4 (24)	0 (0)	6 (35)	1 (33)	8 (47)
Khrucharoen et al. (2019) [13]	14 (88)	15 (83)	10 (63)	14 (78)	4 (25)	4 (22)	14 (88)	15 (83)
Do et al. (2013) [14]	12 (100)	4 (100)	8 (67)	1 (25)	N/A	N/A	9 (75)	2 (50)
Fay et al. (2025) [15]	25 (93)	11 (100)	12 (44)	3 (27)	7 (26)	1 (9)	12 (44)	5 (45)
Shin et al. (2021) [16]	24 (100)	26 (100)	12 (50)	14 (54)	N/A	N/A	1 (4)	2 (8)

**Table 3:** Inclusion and exclusion criteria as reported by the included studies

Study	Inclusion criteria	Exclusion criteria
Butz et al. (2024) [12]	- Patients who underwent minimally invasive therapy for MALS (laparoscopic or robotic)	- Those not eligible for a minimally invasive approach - Patients with > 50% stenosis caused by atherosclerosis
Khrucharoen et al. (2019) [13]	- International classification (ICD-10) for coeliac artery compression syndrome and relevant Current Procedural Terminology (CPT) codes for laparoscopic and robotic-assisted procedures	- Those who received previous endovascular treatment - Those who were diagnosed with concomitant intraabdominal/mesenteric vascular diseases - If 1-month post-operative follow up could not be achieved
Do et al. (2013) [14]	- Patients who underwent minimally invasive therapy for MALS (laparoscopic or robotic)	- Not explicitly stated
Fay et al. (2025) [15]	- ICD-10 for MALS, relevant CPT codes for laparoscopic and robotic-assisted procedures - Operative note review	- Those who had an open approach for MALS
Shin et al. (2021) [16]	- Patients who underwent minimally invasive therapy for MALS (laparoscopic or robotic)	- Re-operative MALS cases - Patients under the age of 18 - Concomitant procedures performed at times of MAL release - Patients lost to follow-up

### Operative outcome

#### Conversion to open

**Fig. 2:**
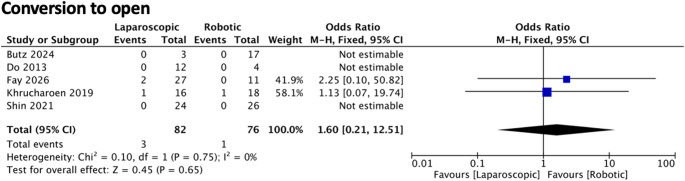
Forest plot of conversion to open

A pooled fixed-effects analysis (laparoscopic *n* = 82, robotic *n* = 76, total *N* = 158) suggested no statistically significant difference in conversion to open between laparoscopic and robotic approaches (OR 1.60, 95% CI 0.21 to 12.51, *p* = 0.65) (Fig. 2). No heterogeneity was observed.

### Short-term postoperative outcomes

#### Postoperative complications

**Fig. 3:**
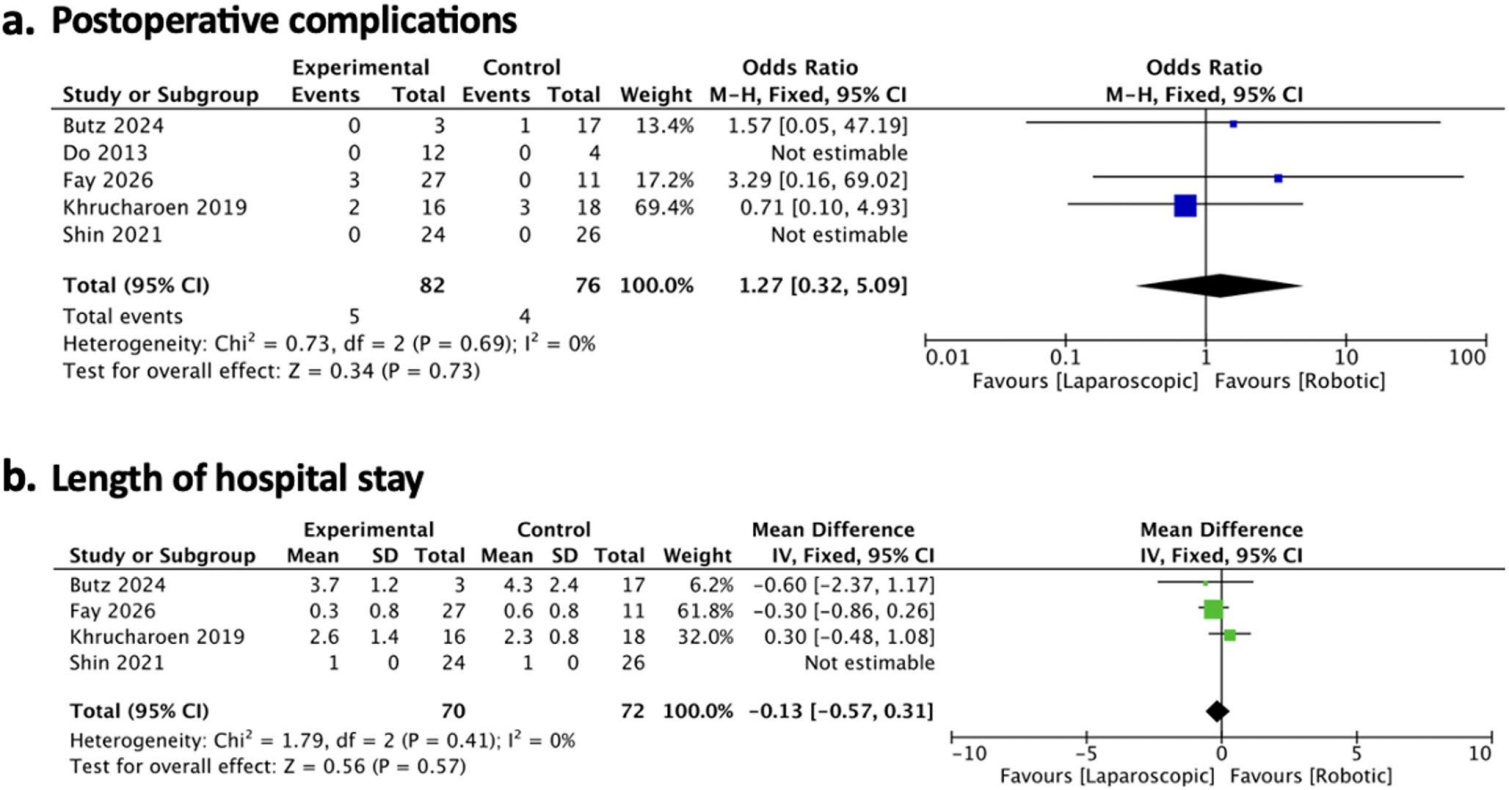
Forest plot of postoperative complications and length of hospital stay

Pooled analyses (laparoscopic *n* = 82, robotic *n* = 76, total *N* = 158) suggested no statistically significant differences between LMALR and RMALR in postoperative complications (OR 1.27, 95% CI 0.32–5.09; *p* = 0.73) (Fig. 3a). No heterogeneity was observed.

### Length of stay

Length of stay was analysed across four studies, with Shin et al. reporting identical lengths of admission between robotic and laparoscopic groups (laparoscopic *n* = 70, robotic *n* = 72, total *N* = 142) [16]. Between the LMALR and RMALR cohorts, the length of hospital admission had no statistically significant difference (MD -0.13 days, 95% CI -0.57 to 0.31, *p* = 0.57) (Fig. 3b). No heterogeneity was observed.

### Symptom outcomes

#### Resolution of pain, symptom recurrence and time to recurrence

**Fig. 4:**
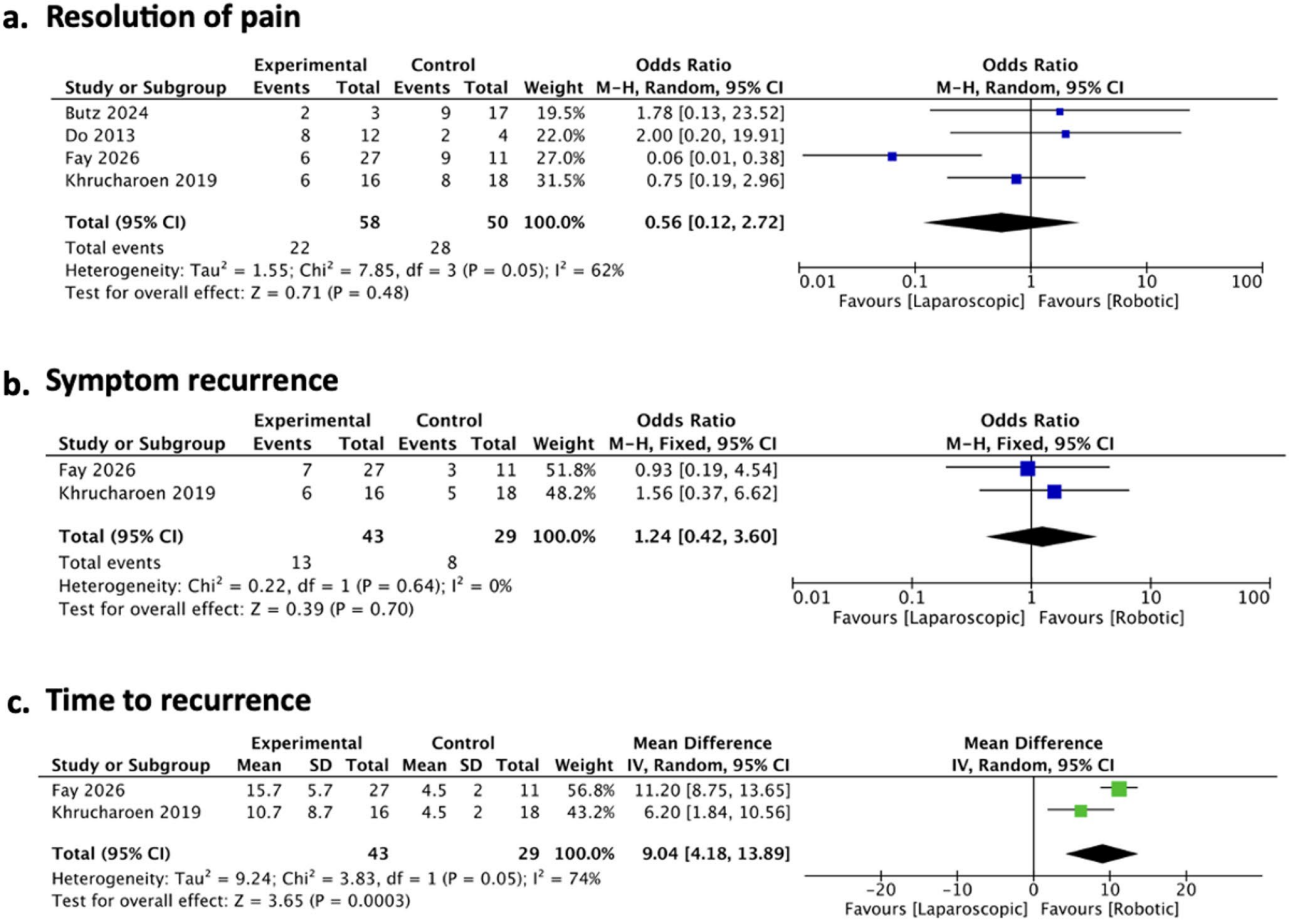
Forest plot of resolution of pain and symptom recurrence and time to recurrence

A pooled fixed-effects analysis (laparoscopic *n* = 58, robotic *n* = 50, total *N* = 108) suggested no difference between the laparoscopic and robotic cohorts in the resolution of pain (OR 0.56, 95% CI 0.12 to 2.72, *p* = 0.48) (Fig. 4a). The substantial heterogeneity (I^2^ = 62%, *p* = 0.05) was calculated. Similarly, no statistically significant difference noted in symptom recurrence (laparoscopic *n* = 43, robotic *n* = 29, total *N* = 92) (OR 1.24, 95% CI 0.42 to 3.60, *p* = 0.70), and there was no heterogeneity between the studies (Fig. 4b). Time to recurrence of symptoms (laparoscopic *n* = 43, robotic *n* = 29, total *N* = 92) was statistically significantly longer in the robotic cohort than in the laparoscopic cohort (MD 9.04, 95% CI 4.18 to 13.89, *p* = 0.0003) (Fig. 4c). Substantial heterogeneity (I^2^ = 74%, *p* = 0.05) was calculated.

#### Postoperative symptoms

**Fig. 5:**
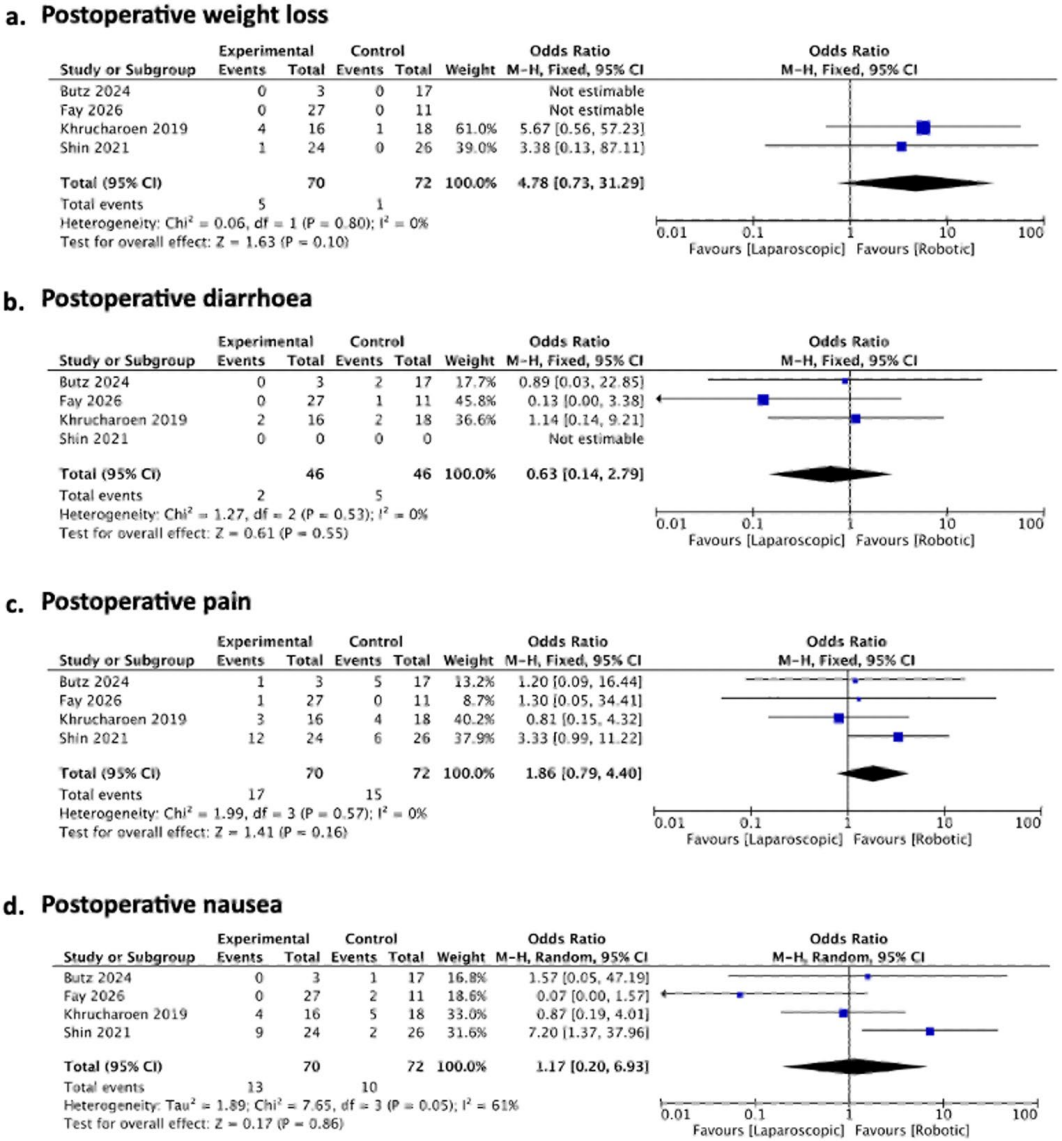
Forest plot of postoperative weight loss, diarrhoea, pain and nausea

Pooled analyses (laparoscopic *n* = 70, robotic *n* = 72, total *N* = 142) suggested no differences between laparoscopic and robotic approaches in weight loss (OR 4.78, 95% CI 0.73–31.29; *p* = 0.10), diarrhoea (OR 0.63, 95% CI 0.14–2.79; *p* = 0.55), or postoperative pain (OR 1.86, 95% CI 0.79–4.40; *p* = 0.16), with no heterogeneity observed for these outcomes (Fig. 5a, b and c). Postoperative nausea (laparoscopic *n* = 70, robotic *n* = 72, total *N* = 142) also did not differ significantly between groups (OR 1.17, 95% CI 0.20–6.93; *p* = 0.86), although substantial heterogeneity was observed (*I*² = 61%) (Fig. 5d).

### Haemodynamic outcomes

Two studies reported preoperative and postoperative coeliac artery PSV. Both studies found postoperative reductions in PSV following decompression, but neither reported a statistically significant difference between laparoscopic and robotic approaches. Because PSV data were limited and reported inconsistently, formal meta-analysis was not performed.

### Quality assessment

Moderate risk of bias was found in four studies and in one study there was low risk of bias according to MINORS. Common methodological limitations across studies included retrospective data collection, absence of prospective sample size calculation, and potential selection bias (Table 4). These limitations were considered when interpreting pooled effect estimates. GRADE demonstrated that all five studies were of low or very low quality. This was largely attributed to the small sample sizes and wide variability in follow-up duration. Refer to supplementary Table 2 for a detailed breakdown of GRADE.

**Table 4:** Quality assessment of the selected studies [12–16]

Author (Year)	Aim	Inclusion of consecutive patients	Prospective data collection	Endpoints appropriate to the aim of the study	Unbiased assessment of endpoints	Follow-up period appropriate to the aim	Loss to follow up < 5%	Prospective calculation of the study size	Adequate control group	Contemporary groups	Baseline equivalence of groups	Adequate statistical analyses	Total MINORS score (out of 24)	Risk of bias
Butz et al. (2024)	2	2	0	2	1	2	1	0	2	2	2	2	18	Moderate
Khrucharoen et al. (2019)	2	2	0	2	1	1	1	0	2	2	2	2	17	Moderate
Do et al. (2013)	2	1	0	2	1	1	1	0	2	1	1	1	13	Moderate
Fay et al. (2025)	2	2	0	2	1	1	1	0	2	2	2	2	17	Moderate
Shin et al. (2021)	2	2	0	2	2	1	2	0	2	2	2	2	19	Low

## Discussion

In this summarised evidence of retrospective comparative studies, laparoscopic and robotic median arcuate ligament release demonstrated similar perioperative outcomes, including conversion to open surgery, postoperative complications, length of hospital stay, symptom resolution, and overall symptom recurrence. A statistically significant difference was observed for time to symptom recurrence, which was longer in the robotic cohort. However, this finding was derived from limited data and should be interpreted cautiously in view of the small sample size, retrospective study design, and heterogeneity in follow-up. The robotic platform may offer technical advantages, including enhanced three-dimensional visualisation, improved instrument articulation, and tremor filtration, which could facilitate dissection around the coeliac axis [15, 16]. One possible explanation for the observed longer time to recurrence is that these technical features aid circumferential dissection of the coeliac artery and more extensive clearance of peri-arterial neural tissue [14, 17]. Several studies noted improved ease of deep dissection around the coeliac axis and reduced reliance on experienced assistants [13, 15, 16]. However, the included studies were not designed to evaluate operative completeness directly, and this interpretation remains speculative.

Robotic procedures were not consistently shorter and were occasionally longer, likely reflecting the additional time required for system setup, docking, and other logistical factors. Nonetheless, increased operative duration may also reflect a more deliberate dissection of the coeliac axis and surrounding neural tissue rather than the logistical demands of the robot alone. Within the included studies, operative time did not appear to correlate consistently with symptom outcomes, suggesting that procedural duration in isolation is unlikely to explain differences in postoperative benefit [13]. Any potential advantage of the robotic platform may therefore relate more to the precision and completeness of dissection than to operative efficiency. The robotic approach may also reduce reliance on a highly experienced first assistant during technically demanding portions of the operation, as highlighted by Shin et al. [16].

Although overall recurrence rates were similar, RMALR demonstrated a longer time to MAL symptom recurrence [13, 15]. The evidence suggests that robotic surgery achieves overall better performance compared to laparoscopy in terms of symptom durability, though this should be interpreted cautiously given the potential for differences in method of follow-up across studies, which is discussed further in the limitations. However, it is worth noting that there was no uniform definition of pain resolution. Variation ranged from either reporting absence or presence of specific symptoms or pain to separately assessing chronic abdominal pain as well as postprandial pain. The heterogeneity in defining resolution of pain, combined with the variable follow-up periods, is a limitation to be considered for future prospective research.

PSV provides an objective haemodynamic marker of coeliac artery decompression; however, the limited available evidence did not demonstrate a clear difference between laparoscopic and robotic approaches in postoperative PSV improvement. Notably, one included study reported greater symptom improvement in the robotic cohort despite a smaller average reduction in PSV [15]. This discordance suggests that clinical benefit in MALS may not depend solely on haemodynamic decompression and may also involve a neurogenic component.

Reintervention outcomes following MAL release were generally poor. Several studies reported patients undergoing additional interventions for persistent or recurrent symptoms, including coeliac artery stenting, balloon angioplasty, coeliac plexus blocks, angiography, and repeat MAL release [12–16]. In Do et al., four patients reported symptom continuation despite a negative DUS [14]. Similarly, in Khrucharoen et al., four patients underwent adjunctive angioplasty with stenting, however, all patients later experienced recurrence of abdominal pain and other symptoms [13]. These poor outcomes further support that vascular stenosis is not the sole contributor to the symptom profile in MALS. This suggests a significant neurogenic component, where compression activates the coeliac ganglion independently of flow limitation through the coeliac artery. Surgical interventions involving celiac ganglionectomy, which is more readily achieved with robotic assistance, may therefore be essential to long-term symptom resolution.

Several limitations should be considered. All included studies were retrospective, sample sizes were small, and outcome definitions and follow-up schedules were not standardised. In addition, robotic cohorts were often treated in more recent time periods than laparoscopic cohorts, introducing potential confounding from learning-curve effects, centre experience, evolving perioperative care, and more intensive follow-up. This is particularly relevant when interpreting symptom recurrence and time-to-recurrence outcomes. Subgroup analysis was considered for results with substantial heterogeneity, however, as only five studies were included, a meaningful subgroup analysis was not possible. For the same reason, assessment of publication bias and sensitivity analyses were not performed. It is also acknowledged that MINORS does not formally evaluate potential confounding or selective outcome reporting bias. As all five studies were retrospective, the possibility of confounding due to selective reporting in desirable outcomes cannot be ruled out and should be considered.

## Conclusion

Our analysis of the available retrospective comparative studies suggests that minimally invasive approaches for (laparoscopic and robotic) MAL release have similar perioperative outcomes and overall recurrence rates. Although robotic surgery was associated with longer time to MAL symptom recurrence, this finding was derived from limited heterogeneous data and should be interpreted cautiously. Prospective multicentre studies with standardised definitions, haemodynamic assessment, and follow-up are required.

## Supplementary Information

Below is the link to the electronic supplementary material.

Supplementary Material 1

## Data Availability

All data analysed in this study are derived from previously published studies. Extracted data and analysis outputs are available from the corresponding author on reasonable request.
