# Supplementary material for: Outcomes in laparoscopic versus robotic-assisted surgery for median arcuate ligament syndrome: a systematic review and meta-analysis
Source: J Robot Surg. 2026 Jun 19;20(1):615. doi: 10.1007/s11701-026-03551-x (PMC13282302; doi:10.1007/s11701-026-03551-x)
Supplement: Supplementary file 1 — Supplementary Material 1 [file 11701_2026_3551_MOESM1_ESM.docx]

Supplementary Table 1. Search strategy

| **Pubmed** |
| --- |
| ((Median Arcuate Ligament Syndrome) OR (Celiac artery compression syndrome) OR (Celiac compression syndrome) OR MALS OR CACS) AND (Laparoscopic OR surgery OR management OR treatment OR operative) |
| **Scopus** |
| ((Arcuate) OR (Celiac) OR MALS OR CACS) AND (Laparoscopic OR surgery OR operative) AND (management OR treatment) AND (syndrome) |
| **EMBASE** |
| ((Median Arcuate Ligament Syndrome) OR (Celiac artery compression syndrome) OR (Celiac compression syndrome) OR MALS OR CACS) AND (Laparoscopic OR surgery OR management OR treatment OR operative) |

Supplementary Table 2. GRADE analysis for quality assessment of each study

| **Study** | **Risk of bias** | **Inconsistency** | **Indirectness** | **Imprecision** | **Publication bias** | **Overall recommendation** |
| --- | --- | --- | --- | --- | --- | --- |
| **Butz et al. (2024)** | Very serious concerns | Moderate concerns | No concerns | Very serious concerns | Some concerns | Very low |
| **Khruch-aroen et al. (2019)** | Serious concerns | Serious concerns | No concerns | Serious concerns | Some concerns | Very low |
| **Do et al. (2013)** | Very serious concerns | Serious concerns | No concerns | Very serious concerns | Some concerns | Very low |
| **Fay et al. (2025)** | Serious concerns | No concerns | No concerns | Serious concerns | Some concerns | Low |
| **Shin et al. (2021)** | Some concerns | Some concerns | No concerns | Some concerns | Some concerns | Low |
